# Durability of Plant Fiber Composites for Structural Application: A Brief Review

**DOI:** 10.3390/ma16113962

**Published:** 2023-05-25

**Authors:** Yunlong Jia, Bodo Fiedler, Wenkai Yang, Xinjian Feng, Jingwen Tang, Jian Liu, Peigen Zhang

**Affiliations:** 1 School of Aerospace and Mechanical Engineering/Aviation, Changzhou Institute of Technology, Changzhou 213032, China; 2 Institute of Polymers and Composites, Hamburg University of Technology, D21073 Hamburg, Germany; 3 Zhejiang Xingyu Autoparts Co., Ltd., Taizhou 317300, China; 4 School of Materials Science and Engineering, Southeast University, Nanjing 211189, China; 5 Wuxi Lintex Advanced Materials Co., Ltd., Wuxi 214145, China

**Keywords:** natural fibers, environment-friendly composites, moisture aging, lightweight design, fatigue

## Abstract

Environmental sustainability and eco-efficiency stand as imperative benchmarks for the upcoming era of materials. The use of sustainable plant fiber composites (PFCs) in structural components has garnered significant interest within industrial community. The durability of PFCs is an important consideration and needs to be well understood before their widespread application. Moisture/water aging, creep properties, and fatigue properties are the most critical aspects of the durability of PFCs. Currently, proposed approaches, such as fiber surface treatments, can alleviate the impact of water uptake on the mechanical properties of PFCs, but complete elimination seems impossible, thus limiting the application of PFCs in moist environments. Creep in PFCs has not received as much attention as water/moisture aging. Existing research has already found the significant creep deformation of PFCs due to the unique microstructure of plant fibers, and fortunately, strengthening fiber-matrix bonding has been reported to effectively improve creep resistance, although data remain limited. Regarding fatigue research in PFCs, most research focuses on tension-tension fatigue properties, but more attention is required on compression-related fatigue properties. PFCs have demonstrated a high endurance of one million cycles under a tension-tension fatigue load at 40% of their ultimate tensile strength (UTS), regardless of plant fiber type and textile architecture. These findings bolster confidence in the use of PFCs for structural applications, provided special measures are taken to alleviate creep and water absorption. This article outlines the current state of the research on the durability of PFCs in terms of the three critical factors mentioned above, and also discusses the associated improvement methods, with the hope that it can provide readers with a comprehensive overview of PFCs’ durability and highlight areas worthy of further research.

## 1. Introduction

Fiber-reinforced polymer composites (FRPs) are highly valued in lightweight designs as they offer exceptional specific properties, including high strength-to-weight and stiffness-to-weight ratios, making them one of the most competitive and significant engineering materials available [1]. They have found broad applications across diverse industries such as aviation, automobiles, wind power, vehicles, sports equipment, etc. [2]. Nevertheless, the extensive utilization of these materials has sparked debates regarding environmental issues [3,4]. Commonly employed reinforcing fibers in FRPs include carbon fibers, glass fibers, and synthetic polymer fibers [5]. These fibers are in essence non-renewable, petroleum-based products whose production processes are highly energy-intensive [6,7]. In addition, the disposal of composites manufactured using these fibers is a matter of concern owing to their limited degradability [8,9]. As a result, the use of plant fibers in structural composites has garnered significant interest as a means to decrease the reliance of the FRP industry on petroleum and lessen their carbon footprint [10,11].

Plant fiber composites (PFCs), as the name suggests, are composites whose reinforcements are fibers extracted from natural plants. Plant fibers (e.g., flax, hemp, sisal, bamboo, etc.) arouse attention in composites not just because they are natural products, but also because they have good specific mechanical properties. Several varieties of plant fibers exhibit similar specific strength and, in some cases, even superior specific moduli, when compared to E-glass fibers [12,13]. Plant fibers offer additional advantages such as low cost (abundant in nature and easy to produce and process), low density, ease of handling, good damping, and non-abrasive properties when compared to petroleum-based fibers [11,13,14,15]. Researchers have proved the promising static mechanical properties of PFCs [16,17]. Recently, PFCs have found practical use in a range of industries including automotive, maritime, sporting goods, transportation, and construction sectors. [18,19]. In most cases, polymers are chosen as the matrix of PFCs. However, the widespread adoption of PFCs in structural components has been limited thus far due to prevailing concerns regarding the long-term durability of these materials [11,20]. The concern lies in two aspects: hydrophilia of plant fibers and insufficient research data on durability of PFCs. The former concern stems from the unique structure and composition of plant fibers, leading to two durability-related challenges with PFCs: the sensitivity of PFCs to moisture and the limited compatibility between hydrophilic plant fibers [21] and hydrophobic polymer matrixes [22]. The second concern necessitates comprehensive research into the durability performance of PFCs under different loading conditions, such as cyclic loads (fatigue), sustained loads over extended periods (creep), progressive loads, and their combinations. Lately, there has been a growing focus on investigating the mechanical properties of PFCs under prolonged cyclic loading conditions [23]. Limited research has been conducted on the creep behavior of PFCs subjected to flexural loads, and these studies have not yielded a thorough understanding of the subject.

To promote the usage of PFCs in structural components or semi-structural components (components that are not load-bearing or structural in nature but still provide some support or functionality to the structure), a clear understanding of the durability of PFCs is needed. Hence, the present review offers an overview of the current status of the research on the durability of PFCs, specifically focusing on what has been relatively extensively reported: the moisture/water aging effects, creep properties, and fatigue properties of PFCs. UV resistance, thermal stability, and antibacterial and antifungal properties are also important factors that affect the durability of PFCs but will not be discussed in this review. Issues related to the above-mentioned three aspects of PFCs are discussed and the corresponding improvement methods are analyzed. Research gaps and promising improvement methods are highlighted.

## 2. Plant Fibers

### 2.1. Fiber Types

**Figure 1:**
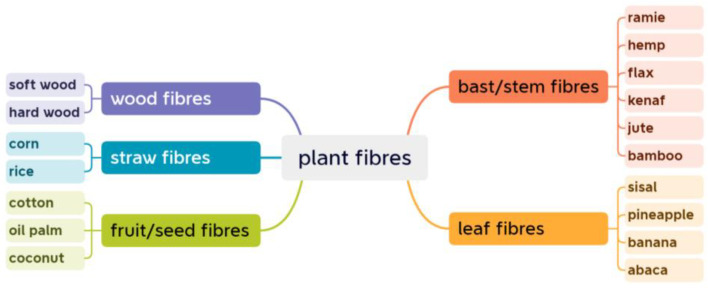
Classification of commonly used plant fibers.

When analyzing durability of PFCs, the exact type of plant fiber is often specified. Commonly used plant fibers in composites can be classified according to their sources [24] (Figure 1). For example, hemp fibers are fibers that are extracted from hemp plants. They can be further classified as bast fibers because they are derived from the inner bark of hemp stems.

### 2.2. Fiber Structure and Composition

The durability of composites heavily relies on the properties of their reinforcements. Therefore, it is important to analyze the unique fiber structure and composition which determine fiber properties.

#### 2.2.1. Fiber Composition

Cellulose, hemicellulose, lignin, and pectin are major constituents of plant fibers [25]. The composition of plant fibers can vary depending on factors such as type of fibers, environmental conditions during growth, plant maturity level, fiber location within plants, and test approaches (see Table 1) [26,27]. The primary constituent of plant fibers is cellulose, which is followed by other basic constituents such as hemicellulose, lignin, and pectin. Some plant fibers also contain other constituents like wax and protein. Among the constituents found in fibers, cellulose stands out as the most rigid and the strongest organic component [28]. However, cellulose is a semi-crystalline polysaccharide that contains numerous polar hydroxyl groups, rendering the amorphous regions of cellulose vulnerable to water molecule absorption [13,20]. Hemicellulose, in contrast to cellulose, is a branched and fully amorphous polysaccharide with a considerably lower molecular weight. It comprises numerous hydroxyl and acetyl groups, contributing to its partial solubility in water and hygroscopic nature [29]. Pectin is a complex polysaccharide that can also attract water. Lignin, characterized by its three-dimensional aromatic structure consisting of phenyl groups, exhibits hydrophobic properties that serve to protect the hydrophilic cellulose and hemicellulose components [30]. The high amount of polar functional groups in the constituents of plant fibers make them hydrophilic, which can raise concerns about the durability of PFCs due to their sensitivity to moisture.

#### 2.2.2. Fiber Structure

In general, plant fibers have the same microstructure despite of the fiber type and species [3]. This makes the analysis easier considering the large range of plant fiber types and species. A single plant fiber is essentially a single long thick-wall cell in plant. It is made up of several concentric layers with a total thickness up to dozens of micrometers. All layers are micro composites reinforced with semi-crystallized cellulose microfibrils which consist of cellulose chains that are highly crystallized and engage in mutual interaction through hydrogen bonding. The size of cellulose fibrils falls within the nanometer range and exhibits variation based on the quantity of cellulose chains present [34]. Each layer differs in the thickness and proportion of constitutive components.

**Figure 2:**
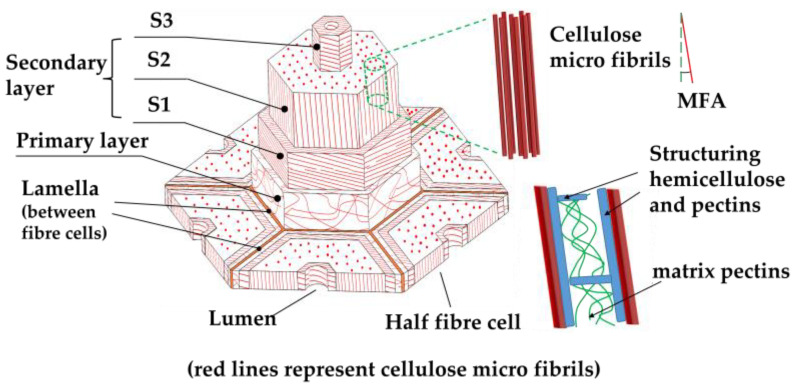
Typical structure of plant fiber (based on the structure of plant fibers [36]).

The microstructure of a typical flax fiber is depicted in Figure 2. The thin first layer, referred to as the P layer, serves as a linkage between the middle lamella and the secondary cell wall (Figure 2). It consists of 3–4 layers of cellulose microfibrils dispersedly embedded in a polymer matrix composed mainly of pectin, hemicellulose, and some proteins [32]. The P layer must remain rigid yet flexible to satisfy two contradictory requirements: to withstand the internal and external stress and to allow cell expansion during plant growth at the same time [34]. The secondary cell wall (S layer) is much thicker than the P layer and is always further divided into three sub-layers (S1, S2, and S3 in radial direction) [35]. Cellulose microfibrils are aligned better in the S layer, with the microfibril angle (MFA, namely the angle between fibrils and fiber axis) often considered to be a crucial structural factor in fiber stiffness and strength. S1 and S3 layers are typically a few micrometers thick with high MFAs. Cellulose micro-fibrils are almost transversely oriented in a matrix composed of hemicellulose and lignin. S1 and S3 play a role in reducing the deformation of S2 when subjected to tension and compression forces. [30]. The S2 layer, with the lowest MFA among the three, makes up for the majority of the fiber’s cross-section and is primarily responsible for the fiber strength and axial stiffness [26]. It is worth noting that all plant fibers have an MFA greater than zero, meaning that the microfibrils in S2 are actually off-axis loaded when fibers are axially loaded. Consequently, PFCs are susceptible to creep under high tensile loads in the fiber direction.

## 3. Moisture/Water Aging Effects

Structural components, particularly those utilized outdoors, frequently face exposure to humid atmospheres, rainfall, or even aqueous environments in real-world scenarios. Over time, PFCs may absorb water under such conditions, leading to a gradual degradation in their mechanical properties. Therefore, it is necessary to evaluate the moisture/water aging effect of PFCs.

### 3.1. Moisture/Water Absorption Mechanism

**Figure 3:**
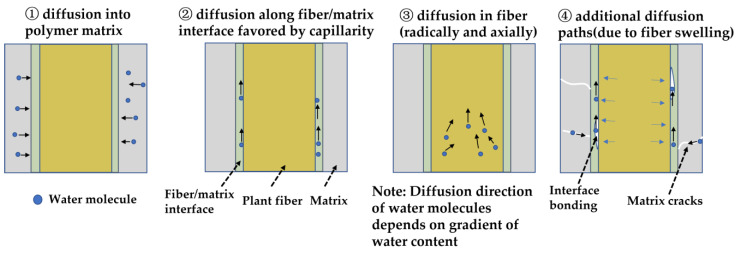
Main moisture/water absorption mechanisms of PFCs (note: the thickness of the interface is exaggerated for illustration).

It is well accepted that moisture/water absorption mechanisms of PFCs are different from that of synthetic fiber composites. Plant fibers not only absorb water molecules, but also act as diffusive paths for water molecules. These damages, in turn, generate additional pathways for water diffusion. Moreover, when plant fibers absorb water molecules, their swelling can result in various forms of damage, such as matrix cracks and fiber-matrix debonding [37,38,39]. These damages, in turn, generate additional pathways for water diffusion. The main mechanisms are explained in literature [40,41], and are depicted in Figure 3. It is worth noting that the dissimilarities in the absorption mechanisms between conditioning environments, i.e., moist or aqueous, are rarely discussed or emphasized in the literature, to the best of our knowledge. However, the primary absorption mechanisms of PFCs are expected to be similar in both of the above-mentioned environments.

### 3.2. Moisture/Water Absorption Behaviour

The typical approach to examining the moisture/water absorption characteristics of PFCs involves assessing the weight gain of conditioned PFC specimens within designated environments. The relative weight uptake of a composite specimen (expressed as a percentage) during wet conditioning can be calculated using Equation (1):

(1) $$M_{t}=\frac{W_{t}-W_{0}}{W_{0}}\times 100,$$

where $M_{t}$ represents the relative weight uptake at a specific time *t*; $W_{t}$ denotes the weight of the wet specimen at time *t*; $W_{0}$ corresponds to the weight of the dry specimen.

In many instances, the global moisture/water absorption characteristics of PFCs can be effectively modeled using diffusion laws. According to diffusion theory, identification of distinct categories of diffusion behavior can be achieved by Equation (2) [42]:

(2) $$\frac{M_{t}}{M_{m}}=k\cdot t^{n},$$

where $M_{m}$ denotes the weight uptake at the saturation state of specimens; k and n are kinetic parameters of diffusion. The exponent *n* is an indication of the diffusion categories. Specifically, when *n* equals 0.5, it signifies the application of Fick’s law. On the other hand, for cases where *n* is not equal to 0.5, it is referred to as non-Fickian diffusion. For instance, when *n* is less than 0.5, the behavior can be classified as pseudo-Fickian, while a value between 0.5 and 1 is considered anomalous diffusion.

Despite the fact that most researchers utilize three-dimensional rectangular PFC specimens for absorption measurements, the simplified Fickian’s second diffusion law (one-dimensional) correlated very well with the measured absorption behavior of PFCs (See Table 2). According to the diffusion law, water uptake increases in a linear fashion with the square root of time in the beginning, and decelerates afterwards until it reaches a steady state. The point distinguishing linear and nonlinear behavior on the absorption curve is mathematically defined as $0.6\cdot M_{m}$. For rectangular specimens, the linear portion of the absorption curve is described as:

(3) $$\frac{M_{t}}{M_{m}}=\frac{4}{h}\times \sqrt{\frac{Dt}{\pi }},$$

where *D* is the diffusion coefficient; *h* is the specimen thickness.

The nonlinear portion, which exhibits weight uptake higher than $0.6\cdot M_{m}$, can be expressed using an approximation equation suggested by Shen and Springer [43]:

(4) $$\frac{M_{t}}{M_{m}}=1-\exp[-7.3{\left(\frac{Dt}{\pi }\right)}^{0.75}],$$

Considering the usage of rectangular specimens according to ISO standards, a geometrical correction factor better reflects the true diffusion coefficient.

(5) $$D_{c}=D_{m}{\left(1+\frac{h}{w}+\frac{h}{l}\right)}^{-2},$$

where $D_{c}$ is the corrected diffusion coefficient; $D_{m}$ is the diffusion coefficient from measured data; l is the specimen length; w is the specimen width.

As a result of the strong attraction of plant fibers to water, PFCs have elevated levels of absorbed water contents when subjected to accelerated aging tests (water baths or high humid environments). $M_{m}$ can reach up to 20% depending on the volume fraction and quality of manufacturing. The majority of reports concerning the diffusion coefficients of PFCs fall within the range of 10^−6^ or 10^−7^ (mm^2^/s).

To have a general impression, moisture/water absorption behavior of a few typical PFCs is listed in Table 2. Owing to the high sensitivity of plant fibers’ properties to water content, the water content of plant fibers ($W_{o}$) prior to manufacturing is especially evaluated for each work.

**Table 2:** Moisture/water aging behavior and the influence on mechanical properties of a few typical PFCs.

Fiber Type, Layout, Matrix, Fiber Volume Fraction (%), Conditioning Environmental	$W_{o}$	$M_{m}(\%)$	$D_{m}$$\;\mathrm{and}\;D_{c}$× 10^−7^ (mm^2^/ss)	Mechanical Properties	Main Conclusions	Work
Flax Unidirectional Epoxy 51% Water bath at room temperature	NA	13.50	$D_{m}$: 13.37 $D_{c}$: 10.51	Tensile strength: reduces by 13% after 1 day and by 15% after 20 days Young modulus: decreases for about 30% after 10 days and for 39% at saturation	Strength and modulus decrease with water absorption. Primary damage mechanism induced by water aging: matrix interface weakening.	[44]
Flax Woven (0°/90°) Bio-epoxy 0.4 and 0.55 55% Deionized water bath, 23 °C, 768 h	NA	8.71 for tensile specimen; 9.76 for flexural specimen	$D_{m}$: 23.2 for tensile specimens;18.5 for flexural specimens	Tensile strength increases by 35%. Flexural strength decreases by 20%. Both tensile and flexural modulus decrease considerably.	Swelling of flax fibers in the composite during water absorption can have positive effects on mechanical properties.	[45]
Flax [0°] and [90°] Bio-based epoxy 40% 80% relative humidity, 30 °C for up to 86 days	~0	5.41 for [0°]; 5.25 for [90°]	$D_{m}$: 2.4 for [0°]; 3.27 for [90°]$D_{c}$: 1.84 for [0°]; 2.74 for [90°]	[0°]: Tensile strength decreases by 5.7% after 5 days, then increases by 18.7% after 35 days. Tensile strength after 86 days is still 13.7% higher than that at dry state. [90°]: Tensile strength and modulus decrease with water absorption.	Tensile strength in fiber direction is not degraded by moisture absorption. The trend featured by a first drop followed by an increase is ascribed to the averaging effects of several factors. Fiber-matrix bonding strength decreases after moisture absorption.	[46]
Hemp Needle punched randomly oriented non-woven Unsaturated polyester 26% Water bath for 888 h	NA (dried)	10.972	NA	The ultimate tensile stress of wet samples is higher than that for dry samples.	The improvement in tensile strength can be ascribed to the swelling of the fibers after water absorption, which could fill the gaps between the fiber and the polymer matrix.	[38]
Kenaf (yarns) NA POM 20 wt.% Cycled tests for 672 h: 45 min UV light + 1 h water spray at 60 °C + moisture relative humidity 70% for 1 h	NA	NA	NA	Tensile strength and flexural strength are reduced by 50% and 18%, respectively, after 672 h accelerated weathering.	The reduction is attributed to the degradation of cellulose, hemicellulose, and lignin content of the kenaf fiber resulting from exposure to moisture and UV.	[47]
Sisal Short fibers (15 mm) Epoxy 20 wt.% Water bath at 30 °C for 15 days	NA	2.86	$D_{m}$: 44.2 for [0°]; 3.27 for [90°]$D_{c}$: 1.84 for [0°]; 2.74 for [90°]	Due to water absorption, there were 13%, 10% and 16% reduction in tensile strength, flexural strength, and impact strength, respectively.	Possible reason for degradation in mechanical properties: swelling of fibers by water absorption resulted in a decrease in stiffness of fibers and an increase in micro-cracks.	[48]
Bamboo Unidirectional Epoxy 42 Water bath at 100 °C for 1 h, 2 h, 3 h, and 4 h	NA	18.97	NA	Tensile strength and flexural strength both decrease by over 50% after 4 h in hot water.	Hygrothermal aging has a detrimental effect on the mechanical properties of the bamboo fiber composites.	[49]

### 3.3. Influence on Mechanical Properties

The mechanical properties of PFCs are susceptible to be changed by moisture/water absorption. In most cases, the moisture/water uptake can cause a notable decrease in tensile and flexural properties due to plasticization and the weakening of the fiber-matrix interface [21,50,51]. This is indeed a disadvantage for the application of PFCs. However, some researchers reported contradictory results (Table 2) regarding the moisture/water aging effects of PFCs, which can lead to confusion while comprehending their actual impact. Water absorption is found to have beneficial effects on the strength of PFCs due to the swelling of plant fibers because it would fill the gap between fiber and matrix. However, the beneficial effects of fiber swelling have not yet come to a consensus since some researchers claim that swelling would lead to micro-cracks at the matrix interface. Antoine le Duigou et al. [52] specially investigated the hygroscopic expansion of plant fibers and found that fiber swelling controls the stress state at the fiber/matrix interface and thus the performance. They believed that the moisture sensitivity of plant fibers could be turned into an advantage if radial stress could be generated. Increasing humidity and moisture content will increase radial stress and thus promote stress transfer at interfaces to an unknown limit. This provides an insight to evaluate the disagreement: the water content of plant fiber prior to the manufacturing of PFCs ($W_{o}$ in Table 2), which is neglected but is an important factor controlling the radial stress at interfaces of PFCs during water absorption. Low $W_{o}$ tends to result in an improved load transfer at the fiber/matrix interface after water absorption, whereas high $W_{o}$ is more likely to have detrimental effects on fiber/matrix interface properties.

The significance of $W_{o}$ also stems from the fact that water content can greatly affect plant fibers. Research has shown that flax fibers and its yarns with high water content are stronger than those with low water content [46,53,54]. C. Baley et al. discovered that drying flax fibers led to a considerable degradation in fiber strength [35]. Additionally, D. Zhang et al. [55] investigated the impact of relative humidity at PFCs manufacturing on the interfacial shear strength and flexural properties of flax/unsaturated polyester composites. At 70% relative humidity, the interfacial shear strength exhibited a significant decline, decreasing by over six times at 90% relative humidity. Nevertheless, it was observed that the highest flexural strength was achieved at an optimal relative humidity level of 40%. Abdul Moudooda et al. [56] studied the effects of $W_{o}$ on unidirectional flax fiber/epoxy composites. Although the flexural properties and tensile modulus exhibited a continuous decreasing trend, an optimum water content (fibers conditioned at 50% relative humidity before infusion) corresponded with the highest tensile strength in the fiber direction (6% higher than the worst).

Given these facts, it is necessary to highlight the importance of $W_{o}$ to the durability of PFCs apart from other factors. Although low $W_{o}$ can be helpful to assure a good manufacturing quality by eliminating the evaporation of water during manufacturing, it would amplify the reaction of PFCs in the mechanical properties during water absorption. In other words, there would be an appropriate $W_{o}$ for each specific PFC configuration that can assure an acceptable variation range in mechanical properties after water absorption. Drying plant fibers prior to manufacturing, which is often recommended by suppliers, might not be necessary for the sake of improving stability of mechanical properties of PFCs.

## 4. Creep

Creep plays a crucial role in determining the durability of structural components since they may be subjected to constant loads for long periods in practical cases. However, to the authors’ knowledge, the existing literature lacks adequate data for evaluating the creep behavior of PFCs, especially for tensile creep behavior of long fiber PFCs.

### 4.1. Creep Deformation

Most available research deals with the flexural creep behavior of PFCs. A study conducted by Acha et al. [57] focused on examining the flexural creep behavior of polypropylene composites reinforced with bi-directional jute fibers. The research revealed a clear correlation between creep deformation and the bonding between the fibers and the matrix. An improved interfacial bonding with maleic anhydride-modified polypropylene resulted in lower creep deformation than untreated ones. Time–temperature superposition principle was tried to predict long-term creep deformation but the result was not satisfactory. Amiri et al. [58] utilized the time–temperature superposition principle to assess the long-term flexural creep compliance of flax/vinyl ester composites, though the specific orientation of the flax fibers was not mentioned in their study. The researchers were able to generate smooth master curves for creep compliance; however, it is worth mentioning that these curves have not been experimentally validated. Meanwhile, Jabbar [59] studied the flexural creep behavior of woven jute fabric-reinforced green epoxy composites and investigated the influence of fiber treatments on the creep behavior of composites. It was observed that treatments enhancing the tensile modulus led to improved resistance against flexural creep. The utilization of Findley’s power law model proved to be beneficial in predicting the long-term flexural creep of composites reinforced with jute fibers. When it comes to the tensile creep behavior of polymer-fiber composites (PFCs), there is limited literature available, particularly in relation to the behavior in the fiber direction. Stochioiu et al. [60] observed pronounced creep deformation even for unidirectional PFCs. Creep recovery tests revealed the existence of plastic deformation. Benjamin Sala et al. [61] conducted short-term creep-recovery tests (2 h in total) on unidirectional flax and stain woven hemp-reinforced epoxy composites. They found that both types of composites exhibited perceivable creep in the load direction, even for the unidirectional ones. Composites conditioned in 70 °C and 85% relative humidity demonstrated higher creep deformation and residual strain than the ones in 23 °C and 50% relative humidity. In our previous work, we also observed pronounced creep for unidirectional flax/epoxy composites even at low stress levels. Improving fiber-matrix bonding can significantly reduce creep deformation. However, it was often not observed that unidirectional glass/carbon fiber composite loaded in the direction of the fibers exhibited visible creep deformation at low levels of creep stress [62]. The reason is ascribed to the arrangement of fibrils in S2 layer of single plant fibers, which are aligned off-axis (see MFA in Table 1). The absence of such off-axis alignment in single glass/carbon fibers explains why visible creep deformation is absent in unidirectional glass/carbon fiber composites loaded in the direction of the fiber. In addition, plant fibers themselves are made of polymers. Therefore, they are found to demonstrate viscoelastic properties even in the axial direction. Based on the limited data on creep of PFCs, some findings on the creep deformation can be summarized:

- PFCs exhibit pronounced deformation under constant load even at low stress levels.
- Suitable models for the prediction of deformation of PFCs in the long-term are still questionable.
- Creep deformation increases with stress levels and can be enhanced in hydrothermal environments or by heat [63].
- Improving fiber-matrix bonding can effectively improve the creep resistance of PFCs.

### 4.2. Creep Rupture Life

**Figure 4:**
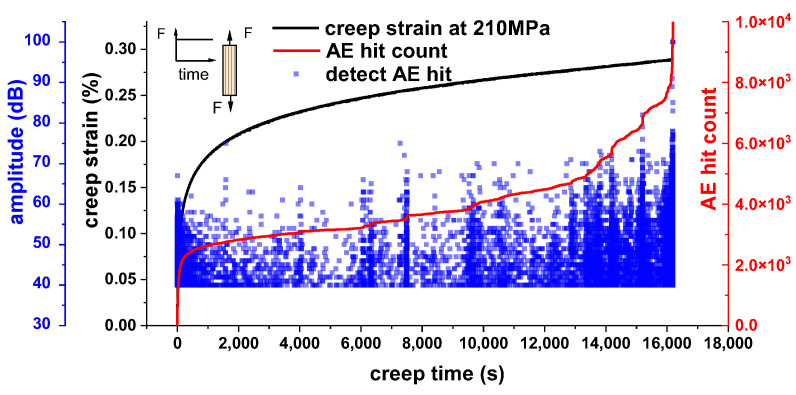
Detected AE signals indicate an intensive damage development during creep of unidirectional flax/epoxy composite at 210 MPa (70% UTS) [62].

Apart from deformation, strength degradation of PFCs during creep is also reported. Benjamin Sala et al. [61] discovered that unidirectional flax/epoxy composites tend to fail within an hour under a load level that equals about 75% of the ultimate tensile strength (UTS). Composites reinforced with woven hemp and cross-ply flax even occasionally fail at approximately 55% and 60% of their UTS, respectively. These indicate a relatively fast degradation in strength of PFCs during creep at high stress levels. In our study, we also observed the occurrence of short creep rupture life in PFCs when subjected to high stress levels [62]. Unidirectional flax fiber-reinforced epoxy composites broke within 1 h during creep at 80% of UTS. Even reducing the creep stress to 70% of UTS, all specimens broke after a few hours. The intensive damage development revealed by acoustic emission (AE) detection (Figure 4) and fracture morphology analysis was found to be the primary cause of the strength degradation during creep.

## 5. Fatigue

Fatigue performance is an important design criterion for engineering materials. This work exclusively focuses on the research of fatigue endurance and life prediction of PFCs. Fatigue behaviors (such as damage mechanisms, changes in strength and stiffness, etc.) of PFCs have been well reviewed in literature [23,64], and hence are not discussed in this work.

### 5.1. Fatigue Endurance

The stress-life method, commonly characterized by a stress-number of cycles (*S*-*N*) curve (also known as a Wöhler curve) is often used to evaluate the fatigue life of PFCs. The curve is obtained by plotting applied stress (*S*) against component life or number of cycles to failure (*N*). A summary of the fatigue strengths of typical PFCs is provided to gain an overall understanding of the fatigue performance discussed in existing publications (Table 3). Fatigue strength is defined in this study as the maximum stress level at an endurance of one million cycles. As seen in Table 3, aligned unidirectional PFCs exhibit the highest fatigue strength at the endurance limit, primarily attributed to their superior static strength. In contrast, other PFC types demonstrate a decreasing trend in fatigue strength, ranging from cross-ply [0°/90°], quasi-isotropic, twill-woven reinforced, [±45°], random-oriented mat reinforced, to transverse direction [90°] configurations. The majority of investigated PFCs, regardless of plant fiber type and textile architecture, can survive over one million cycles at a maximum tensile stress level lower than 40% UTS. This indeed boosts confidence in the application of PFCs in structural applications. More encouragingly, PFCs with certain textile architectures exhibit a lower fatigue degradation rate than that of glass-fiber-reinforced composites (GFRPs). Shah et al. [65] carried out tension-tension fatigue tests on unidirectional flax fiber composites and observed a slower rate of damage development and fatigue strength degradation. This was evident from the *S*-*N* curve of flax fiber composites, which had a less steep slope compared to that of GFRPs. Similarly, Bensadoun [66] found that composites reinforced with three types of flax fabrics (random mat, twill 2 × 2, and quasi-unidirectional [0°,90°]) exhibited a slower rate of fatigue strength degradation and a more stable stiffness evolution compared to composites with the same layout of glass fibers. Liang et al. [67] compared fatigue properties of flax fiber composites and glass fiber composites with the two layouts: cross-ply [0°/90°] and angled cross-ply layout [±45°]. They confirmed a slower rate of fatigue strength degradation for both layouts compared to GFRPs as well.

### 5.2. Fatigue Life Prediction

The accurate prediction of fatigue life of PFCs has not yet been achieved due to the complex fatigue damage development and the high fatigue sensitivity. Nonetheless, reasonable predictions on fatigue life of PFCs at a given stress ratio are often done by analyzing the *S-N* curve of PFCs. Most researchers tended to use a linear relationship to fit the *S-N* curves of PFCs, while some found the power function was suitable for fitting [65]. Whichever is more suitable for PFCs is hard to deduce from currently available data, yet either should give reasonable prediction on fatigue life as long as experimental data are sufficient to generate an *S-N* curve. *S-N* curves can be further used to present data in the more convenient format: constant-life diagrams. Constant-life lines enable fatigue lives to be predicted at any stress ratio. Mean stress (*x* axis) is plotted versus stress amplitude (*y* axis) and for any number of fatigue cycles to failure. Towo et al. [71] developed constant-life diagrams for sisal fiber composites by fitting a third-order polynomial curve through the data points derived from *S-N* curves at *R* = 0.1 and *R* = −1, along with the static tensile and compressive strengths. Shah et al. [65] generated a complete Haigh constant-life diagram using data obtained from the power–law regression lines of the *S-N* diagrams for hemp/polyester composite specimens tested under the five different stress ratios. They also applied this constant-life diagram for the fatigue design and life prediction of a 3.5-m long hemp/polyester wind turbine blade [76]. However, it should be noted that obtaining sufficient fatigue data for several *R* ratios to ensure the accuracy of constant-life diagrams can be costly and time-consuming.

Apart from analyzing *S-N* diagram, there are other proposed methods for predicting fatigue life of PFCs. Sawi et al. [77] performed fatigue tests on flax/epoxy composites with two configurations ([0°] and [±45°]). They found *S-N* curves of both composites exhibit a linear relationship when plotted in a semi-log scale. They also provide a method to determine the high cycle fatigue strength of PFCs by plotting the average stabilized temperature of tested PFCs against applied stress levels. However, it seems this method has not been widely adopted by other researchers yet. Ahmed Fotouh et al. [74] developed a mathematical model to predict the fatigue life of PFCs. The model was derived from a Norton–Hoff rheology model for viscoplastic material and incorporates a new modified stress level, which was used to normalize all fatigue life curves for different fatigue loading and environmental conditions. Experimental results showed that the model was capable of predicting the fatigue life of the tested hemp fiber composites with different fiber volume fractions (*Φ*) and stress ratios (*R*), as well as taking into consideration the effect of moisture absorption. However, the model is established based on the matrix-dominated fatigue behavior. Hence, it is only applicable to PFCs which have matrix-dominated fatigue behaviors, e.g., short fiber-reinforced composites.

## 6. Towards Improving the Durability

### 6.1. Towards Improving Moisture/Water Aging

The moisture/water absorption of PFCs can be reduced by several methods. Chemical treatments, sol-gel coating, liquid flame spray nanocoating, dip coating, single-step deposition, etc. have been reported effective in increasing the hydrophobicity of plant fiber surfaces. Details of these approaches were well reviewed in works [11,20,78], and hence are not discussed in this work. Some of these approaches are reported beneficial in reducing moisture/water absorption of PFCs [79]. Marie Bayart et al. [80] coated colloidal silica fume on flax fibers and compared the moisture absorption resistance of composites made with treated and untreated fibers. The treated composites showed less severe degradation of mechanical properties after conditioning in a deionized water bath (50 °C), with higher retention rates for Young’s modulus (43.3% vs. 50.8%) and ultimate tensile strength (61% vs. 50.3%). Comparatively, the maximum water absorption was only slightly lower for the treated composites (11.17%) than for the untreated ones (12.45%). Maruyama, S. et al. [81] studied the effect of silane treatment on the water absorption and mechanical properties of polylactic acid/short bamboo fiber (PLA/SBF) composites. The results showed that treating bamboo fibers with silane can enhance the water absorption resistance of the composites. After undergoing 250 h of conditioning in a distilled water bath, the tensile strength of the silane-treated and untreated composites experienced reductions of 55% and 45%, respectively. Notably, the maximum water absorption of the composites did not decrease significantly after silane treatment. Ameer and his team [82] developed a hydrophobic treatment for jute fibers, involving scouring, mercerization, and hydrophobic finishes. The treated jute/unsaturated polyester composites showed a maximum water absorption of 14.9%, a 40% improvement compared to the untreated ones which had a maximum water absorption of 25%. The flexural strength degradation rate of the treated composites was approximately 24% slower than that of the untreated ones.

Apart from treatments on plant fiber surfaces, there are other simple but effective ways to alleviate the moisture/water absorption of PFCs—hydrophobic coatings on PFCs surfaces. Mokhothu and John [83] investigated the application of a poly furfuryl alcohol-based coating on flax fabric reinforced phenolic panels. After conditioning at 90 °C and 90% relative humidity for three days, the coated samples showed a 75% reduction in moisture absorption and a 21% and 16% decrease in tensile modulus and tensile strength, respectively. Liu and Tisserat [84] coated flax fiber composites with acrylated epoxidized soybean oil and found a 30% reduction in water uptake than their uncoated counterparts. For now, it is difficult to determine which methodology (fiber surface treatment or bio-based coating on PFCs surfaces) is superior for improving moisture/water aging of PFCs due to insufficient data. However, we believe that applying a bio-based coating to composite surfaces may be a more feasible industry solution than treating plant fiber surfaces, due to its effectiveness, low cost, and ease of handling.

Moreover, it is important to note that the current proposed approaches cannot completely eliminate the moisture/water absorption of PFCs as it is impossible to remove all polar groups from plant fibers. In other words, the moisture/water absorption issue of PFCs can only be alleviated unless all polar groups of plant fibers are removed or modified. For example, we found that pre-coating flax fibers with polymerized furfuryl alcohol can considerably lower the moisture/water absorption rate but have no apparent reduction in maximum water content after three months’ conditioning [36].

### 6.2. Towards Improving Creep

Several pieces of research have demonstrated that improving fiber-matrix bonding can effectively reduce creep defamation of PFCs. Acha et al. [57] found that low creep deformation of jute/polypropylene composites correlated with the ones with improved fiber-matrix bonding. Amiri et al. [58] used alkaline to treat flax fibers and found that the treatment delayed the creep response and slowed the process of creep in flax/composites in the steady state region. The creep compliance of the treated composites is approximately 10% lower than that of the untreated ones at 10^8^ s and the advantage gradually expands with time (according to predicted creep compliance master curves). The reason was ascribed to an improved fiber-matrix bonding as well. Militký and Jabbar [85] compared short-term flexural creep of jute fiber-reinforced composites whose reinforcements had undergone infrared laser, ozone, enzyme, and plasma treatments. The results showed that all treated composites exhibited less creep strain than untreated ones at all temperatures. Laser-treated composite revealed the best interlocking of fibers and matrix at the interface, and therefore exhibited the least creep deformation (32%, 48%, and 81% lower at 40 °C, 70 °C, and 100 °C, respectively, under a flexural stress of 2 MPa compared to the untreated ones). In our previous work, pre-coating flax fiber with polymerized furfuryl alcohol (FA) lead to considerable creep deformation. The initial strain and overall tensile creep strain of FA-treated composites at 132 MPa were on average 19% and 34% lower than those of untreated ones, respectively. The reduced creep after FA treatment can be ascribed to an improved inter-fiber and fiber-matrix bonding, which constraints better the deformation of flax fibers caused by reorientation effects. Furthermore, much less damage (mainly refer to matrix cracks and fiber-matrix debonding) was found for FA-treated composite than untreated ones, evidenced by AE detection [62]. Although data are not sufficient for now, it can be seen that improving fiber-matrix bonding can effectively reduce creep defamation of PFCs. It should be noted that creep cannot be eliminated, unless the plant fiber structure is modified to completely eliminate MFA of fibers.

### 6.3. Towards Improving Fatigue

For now, the improvement of fatigue performance of PFCs has not gained as much attention as other aspects of PFCs. Nonetheless, recent available research work has revealed a general trend that good fiber/matrix adhesion can lead to higher long-term durability [86,87]. Asumani and Paskaramoorthy [88] performed two fiber treatments (alkali-alone treatment and combined alkali-silane treatment) on kenaf fibers and tested the fatigue performance of kenaf-reinforced polypropylene composites. Both fiber treatments can improve the fatigue of the composites, with alkali-silane treatment being the most effective one. Microscopy examination indicated apparently improved fiber-matrix adhesion by alkali-silane treatment. Kenaf fiber composites treated with 35 wt.% alkali-silane (4% concentration) sustained an average of 64% more fatigue cycles to fracture at 45% of their UTS than untreated composites. A similar finding was reported by Muessig et al. [89], who promoted the bonding between regenerated cellulose fibers and polypropylene matrix via photochemical surface modification of the fiber. Composites with 1% pentaerythritol triacrylate treated regenerated cellulose fibers exhibited an approximately 18% increase in fatigue strength at one million cycles compared to untreated composites. The correlation between improved fiber-matrix bonding and enhanced fatigue strength of PFCs is reasonable, for fiber-matrix debonding is believed to be one of the major damage mechanisms of PFCs during fatigue loading [23]. Hence, it is highly possible that treatments that improve fiber-matrix bonding would be beneficial for fatigue performance of PFCs. Besides the fiber-matrix bonding, the strength of the composite affects durability. It has been shown in Table 3 that the fatigue strength of PFCs is somehow proportional to their UTS, which means composites with higher static strength are generally more durable. Therefore, it can be deduced the methods that can significantly increase the UTS of PFCs would very likely result in prolonged fatigue life.

### 6.4. Further Improving—Promising Methods

As discussed above, it seems treatments which can promote fiber-matrix bonding are helpful for improving durability of PFCs. However, more work needs to be done due to the insufficient data, especially the lack of data about the effects of treatments on creep resistance and fatigue performance of PFCs. Apart from that, attention should also be drawn to further improve the durability of PFCs, since the improvement made by conventional treatments could be limited due to the chemical composition and microstructure of plant fibers. On the one hand, polar groups in plant fibers cannot be fully eliminated by treatments, which limits the improvement in moisture/water aging. On the other hand, conventional treatments cannot change the fact that cellulose fibrils are off-axis aligned to fiber axis, which limit the improvement in creep and possibly in fatigue performance.

**Figure 5:**
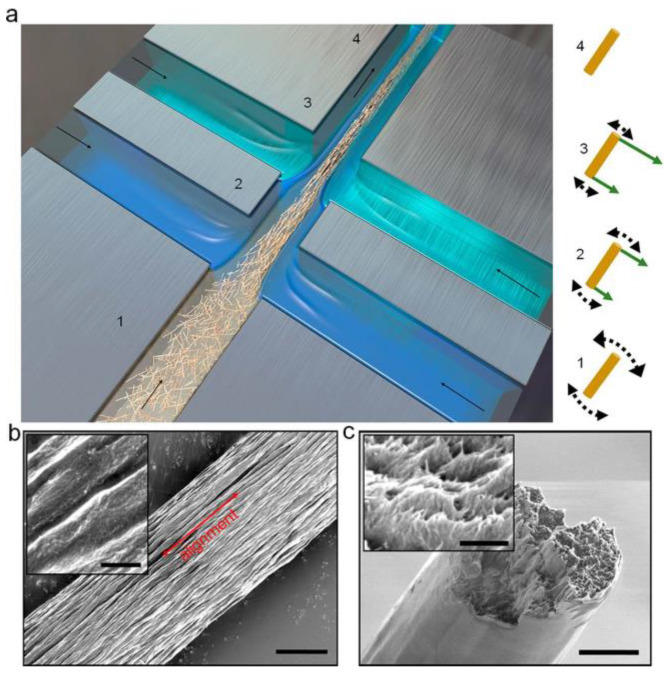
Production and morphology of continuous cellulose fiber made of cellulose nano fibers: (**a**) Schematic of hydrodynamically aligning and assembling CNFs (MFA is nearly eliminated); (**b**) SEM image of the fiber surface; (**c**) SEM image of the cross-section of the fiber. The scale bars in (**b**,**c**) measure 3 μm, while the insets are 400 nm in size. [94].

Reconstruction of cellulose fiber using cellulose nanofibers (CNFs) seems a promising approach to further enhance the durability of PFCs. Cellulose nanofibers are abundant in plant fibers and have much higher theoretical strength (1–3 GPa) and theoretical modulus (130–150 GPa) than plant fibers [90], making them promising building blocks for the high-performance cellulose fibers [91]. Recently, continuous and lengthy macro-fibers made of CNFs have been successfully constructed by researchers [92,93,94,95,96]. Hakansson et al. [92] devised a method that is continuous and has the potential for industrial scalability, possible to produce robust and rigid cellulose filaments by hydrodynamically arranging and assembling CNFs. Mittal et al. [94] produced perfect unidirectional macroscale cellulose fibers through a flow-assisted organization of CNFs (Figure 5). Young’s modulus and tensile strength of the reconstructed continuous cellulose fiber reach as high as 86 GPa and 1.57 GPa, respectively. It can be seen that composites made by these man-made macro cellulose fibers are very likely to have further improved fatigue strength due to the increase in UTS and significantly enhanced creep resistance due to the elimination of MFA.

**Figure 6:**
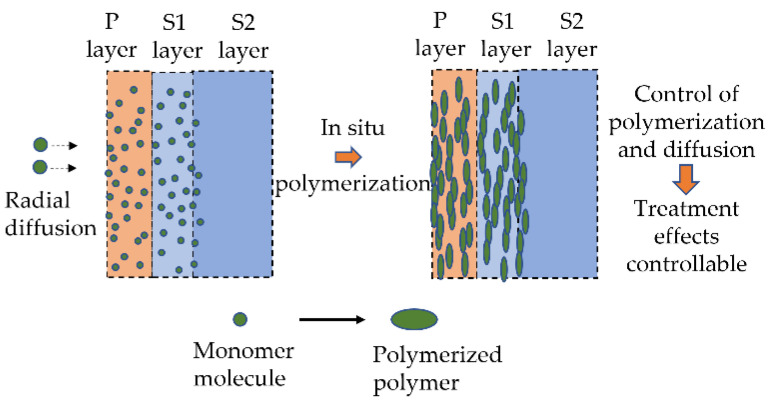
Illustration of cell wall engineering using small monomer molecules to achieve inner treatment on plant fibers.

Apart from reconstructing chemical compositions and microstructures of plant fibers, novel fiber treatment that can reach the inner part of plant fibers might also be promising. Li, Zhang et al. [91,97,98,99] conducted a set of research that reveals multi-stage interfacial failure behaviors of sisal fiber composites due to the hierarchical microstructure of plant fibers, implying the possible important role of interfaces inside plant fiber in improving durability of PFCs. On that account, we proposed a possible approach of fiber treatment which could reach the inner part of plant fibers. The idea (depicted in Figure 6) is based on the fact that small molecules can diffuse through plant fibers. If small monomer molecules are used for diffusion and in situ polymerized inside plant fibers afterward, inner treatment on plant fibers can be reached. By changing the amount of monomer and diffusion depth, one can tailor the treatment effects. However, the challenge would lie in finding the appropriate monomers.

## 7. Conclusions and Prospects

This work reviews the research on the moisture/water aging effects, creep properties, fatigue properties of PFCs, and the corresponding improvement methods. We also discuss new methods and concepts that may yield promising results. Some key points on the durability of PFCs are summarized as follows:

1. Diffusion coefficients of PFCs reported in literature are in the range of 10^−6^ or 10^−7^ (mm^2^/s), and PFCs are sensitive to moisture/water absorption. Initial drying of plant fiber is not necessary if one wants to have a better stability in mechanical properties of PFCs upon water/moisture absorption. In general, the absorption of moisture or water can significantly diminish the tensile and flexural properties of materials, primarily because of the plasticization effect and the deterioration of the fiber-matrix interface. Currently proposed approaches, such as fiber surface treatments and coatings, are able to alleviate the impact of water absorption on the mechanical properties of PFCs, but complete elimination seems impossible, thus limiting the application of PFCs in moist environments. Applying bio-based coatings to composite surfaces may be a more feasible industry solution than treating plant fiber surfaces, due to its effectiveness, low cost, and ease of handling.
2. Creep of PFCs is found noticeable at both low and high stress levels possibly due to off-axis aligned cellulose fibrils. Increasing fiber-matrix bonding proves very effective to improving creep resistance of PFCs but extensive research on this topic is still needed. However, creep cannot be eliminated unless the plant fiber structure is modified to completely eliminate MFA of fibers.
3. Most research focuses on tension-tension fatigue properties, but data on compression-related fatigue properties are limited. PFCs have demonstrated a high endurance of one million cycles under a tension-tension fatigue load at 40% of their ultimate tensile strength (UTS), regardless of plant fiber type and textile architecture. *S-N* curve gives reasonable prediction on fatigue life of PFCs, but accurate prediction has not yet been achieved due to the complex fatigue damage development and the high fatigue sensitivity. While recent research has indicated that good adhesion between fibers and the matrix can enhance the long-term durability of PFCs, more evidence is needed to validate this trend.
4. Continuous cellulose fibers made of CNFs that are much stronger than plant fibers might bring further improvement in durability. Fatigue strength is very likely to be further improved due to an increase in UTS, and creep resistance would be improved due to the elimination of MFA. Cell wall engineering on plant fibers which can modify the inner part of plant fibers might also be promising because that could improve the multi-stage interfacial failure behaviors of PFCs.

Overall, PFCs have been shown to have good durability. They can be used for commercial products such as automobile parts (door panels, dashboards, and interior trims), building materials (such as roofing tiles, flooring, insulation, and wall panels), consumer goods (such as furniture, toys), and outdoor sports goods (such as bicycle frames, snowboards, skis, surfboards, helmets), provided measures such as coatings or fiber treatments can be applied to alleviate the moisture/water absorption and creep. Moreover, it is believed that more commercial applications for PFCs will emerge as their durability is better understood, and as fiber production and treatment technologies are developed further.

## Figures and Tables

**Table 1:** Chemical composition and some physical properties of common plant fibers (data are summarized from [12,31,32,33]).

Fiber Type	Composition						Physical Properties	
	Cellulose (wt.%)	Hemicellulose (wt.%)	Lignin (wt.%)	Pectin (wt.%)	Wax (wt.%)	Moisture (wt.%)	MFA * (deg)	Length (mm)
Abaca	56–63	15–17	7–9	1	3	5–10	22.5	
Bamboo	26–65	30	5–31	-	-		2–10	1.5–4
Banana	63–67.6	6–19	5–10	3–5	-	8.7–12	11–12	300–900
Coir/Coconut	32–43.8	0.15–20	40–45	3–4	-	8	30–49	20–150
Cotton	82.7–90	5.7	0–5	0–1	0.6	7.85–8.5		10–60
Flax	62–75	11–20.6	2–5	1.8–2.3	1.5–1.7	7.9–10.0	5–10	5–900
Hemp	68–74.4	15–22.4	3.7–10	0.9	0.8	6.2–12	2–6.2	5–55
Jute	61–71.5	13.6–20.4	11.8–13	0.2–0.4	0.5	12.5–13.7	8	1.5–120
Kenaf	31–72	20.3–21.5	8–19	3–5	-	8.35	-	2–61
Oil palm	60–65	-	11–29	-	-	-	42–46	
Ramie	68.6–85	13–16.7	0.5–0.7	1.9	0.3	7.5–17	7.5	12–15
Sisal	60–78	10–24	8–14	10	2	10–22	10–22	900
Softwood **	40–45	25–30	26–34	-	-	-	5–30	2–6
Hardwood **	45–50	21–35	22–30	-	-	-	5–30	1–2

* MFA represents the angle between the fiber axis and the fibrils within the second fiber layer. ** Contents of extractives in softwood fibers and hardwood fibers are 0–5% and 0–10%, respectively.

**Table 3:** Overview of fatigue endurance of PFCs in literature.

Fiber/Matrix	Textile Architectures/ Laminate Configurations	Fiber Volume Fraction (%)	UTS (MPa)	Fatigue Test Conditions ^1^	Fatigue Strength at 10^6^ Cycles to UTS ^2^	Work
Flax/polyester	[0°]	27.7	164.3	10 Hz; *R* = 0.01	(0.45, 0.5)	[65]
Flax/polyester	[±45°]	28.9	073.7	5 Hz; *R* = 0.1	0.4	[65]
Flax/epoxy	[0°/90°]	43.7	170 ± 19.6	5 Hz; *R* = 0.1	0.4	[67]
Flax/epoxy	[±45°]	42.5	079 ± 6.6	5 Hz; *R* = 0.1	(0.5, 0.6)	[67]
Flax/epoxy	Random mat	30	084	5 Hz; *R* = 0.1	0.3	[66]
Flax/epoxy	Low twist twill	40	120	5 Hz; *R* = 0.1	0.35	[66]
Flax/epoxy	Twill weave	34.3	106 ± 2.9	5 Hz; *R* = 0.1	Ca. 0.4	[68]
Flax/epoxy	Quasi-isotropic	NA	145.6 ± 7.2	5 Hz; *R* = 0.1	(0.5, 0.6)	[69]
Flax/epoxy	[90°]	43.1	026.1 ± 0.6	5 Hz; *R* = 0.1	(0.4, 0.5)	[70]
Flax/epoxy	[0°]	43.1	318 ± 12	5 Hz; *R* = 0.1	0.4 or lower	[70]
Flax/epoxy	[0°]	40	300.3 ± 7.1	5 Hz; *R* = 0.1	0.4 or higher	[36]
Sisal/polyester	[0°]	68–72	223	400 MPa/s; *R* = 0.1	Ca. 0.45	[71]
Sisal/epoxy	[0°]	68–72	329	400 MPa/s; *R* = 0.1	Ca. 0.47	[71]
Sisal/epoxy	[0°]	68–72	329	400 MPa/s; *R* = −1	Ca. 0.15	[71]
Sisal/epoxy	Random short fiber	35	044.8	5 Hz; *R* = 0.01	0.45	[72]
Hemp/epoxy	[0°/90°]	36 ± 2	113 ± 9	1 Hz; *R* = 0.01	0.4 or higher	[73]
Hemp/epoxy	[±45°]	36 ± 2	066 ± 7	1 Hz; *R* = 0.01	0.45 or higher	[73]
Hemp/HDPE	Chopped fibers	13.5	029.54 ± 0.18	3 Hz; *R* = 0.1	Ca. 0.4	[74]
Jute/polyester	[0°]	31.7 ± 0.1	175.1 ± 10.3	10 Hz; *R* = 0.1	Ca. 0.49	[65]
Kenaf/epoxy	[0°]	45	100.56	5 Hz; *R* = 0.5	Ca. 0.5	[75]

^1^ Fatigue tests were conducted under cyclic loads with constant stress amplitude, while fatigue loads in work [71] were applied at a constant stress rate. “*R*” refers to stress ratio which is the ratio of the minimum stress to maximum stress. ^2^ Most values were not given directly by authors and were estimated via the regression line/curve from the *S-N* diagrams illustrated by the authors.

## Data Availability

Not applicable.
